# Unclear tumor border in magnetic resonance imaging as a prognostic factor of squamous cell cervical cancer

**DOI:** 10.1038/s41598-023-42787-7

**Published:** 2023-09-16

**Authors:** Mamiko Sato, Satoshi Tamauchi, Kosuke Yoshida, Masato Yoshihara, Yoshiki Ikeda, Nobuhisa Yoshikawa, Hiroaki Kajiyama

**Affiliations:** Department of Obstetrics and Gynecology, Nagoya University Graduate School of Medicine, 65 Tsuruma-cho, Showa-ku, Nagoya, 466-8550 Japan

**Keywords:** Cancer, Oncology

## Abstract

Magnetic resonance imaging (MRI) is used for pretreatment staging in cervical cancer. In the present study, we used pretreatment images to categorize operative cases into two groups and evaluated their prognosis. A total of 53 cervical cancer patients with squamous cell carcinoma who underwent radical hysterectomy were included in this study. Based on MRI, the patients were classified into two groups, namely clear and unclear tumor border. For each patient, the following characteristics were evaluated: overall survival; recurrence-free survival; lymph node metastasis; lymphovascular space invasion; and pathological findings, including immunohistochemical analysis of vimentin. The clear and unclear tumor border groups included 40 and 13 patients, respectively. Compared with the clear tumor border group, the unclear tumor border group was associated with higher incidence rates of recurrence (3/40 vs. 3/13, respectively), lymphovascular space invasion (24/40 vs. 13/13, respectively), lymph node metastasis (6/40 vs. 10/13, respectively), and positivity for vimentin (18/40 vs. 10/13, respectively). Despite the absence of significant difference in recurrence-free survival (p = 0.0847), the unclear tumor border group had a significantly poorer overall survival versus the clear tumor border group (p = 0.0062). According to MRI findings, an unclear tumor border in patients with squamous cell cervical cancer is linked to poorer prognosis, lymph node metastasis, and distant recurrence of metastasis.

## Introduction

Cervical cancer is one of the most common types of cancer in women. In Japan, approximately 10,000 women develop cervical cancer annually; of those, ~ 3000 women expire due to the disease^1^. Preoperative cervical cancer staging is based on tumor size and the degree of pelvic extension. According to the International Federation of Gynecology and Obstetrics (FIGO) guidelines, staging is based on a combination of physical examination, imaging studies, and endoscopy. Preoperative evaluation is critical because treatment selection is based on its outcome.

Magnetic resonance imaging (MRI) is the most sensitive and specific imaging modality for initial staging and follow-up of cervical cancer^2,3^; this method is widely used in Japan. The updated FIGO 2018 guidelines recommend the use of any imaging modality, pathological findings, or both, for staging^4^. MRI offers high contrast resolution for soft tissue, rendering it a preferred modality for local staging of cervical cancer^5^. Notably, MRI is critical for the treatment of cervical cancer. Specifically, it assists in selection of treatment (e.g., radical surgery, fertility-sparing surgery, concurrent radiotherapy, chemotherapy, and palliative therapy).

Cancer lesions tend to form various types of masses in the cervix (e.g., growing outward, and progressing to the vaginal wall, the parametrium, and uterine corpus). In MRI, such lesions are visualized as T2 hyperintense signals, and are distinguishable from the normal T2 hypointense signal of the fibromuscular stroma^5^. Tumor size is a well-established prognostic factor^6^. However, thus far, there are no reports about the relationship between the clarity of tumor border and the prognosis of cervical cancer and immunohistochemical characteristics.

Vimentin is a marker of epithelial-to-mesenchymal transition (EMT); its expression and functions have been implicated in various types of cancer. In cervical cancer, high expression of vimentin has been associated with lymph node metastasis, lymphovascular space invasion (LVSI), and prognosis^7^. However, the potential association of vimentin expression with MRI findings has not been investigated thus far.

In the present study, we used MRI to categorize cases into two groups, and evaluated the patient characteristics, tumor characteristics, and disease prognosis.

## Materials and methods

### Patients

**Figure 1:**
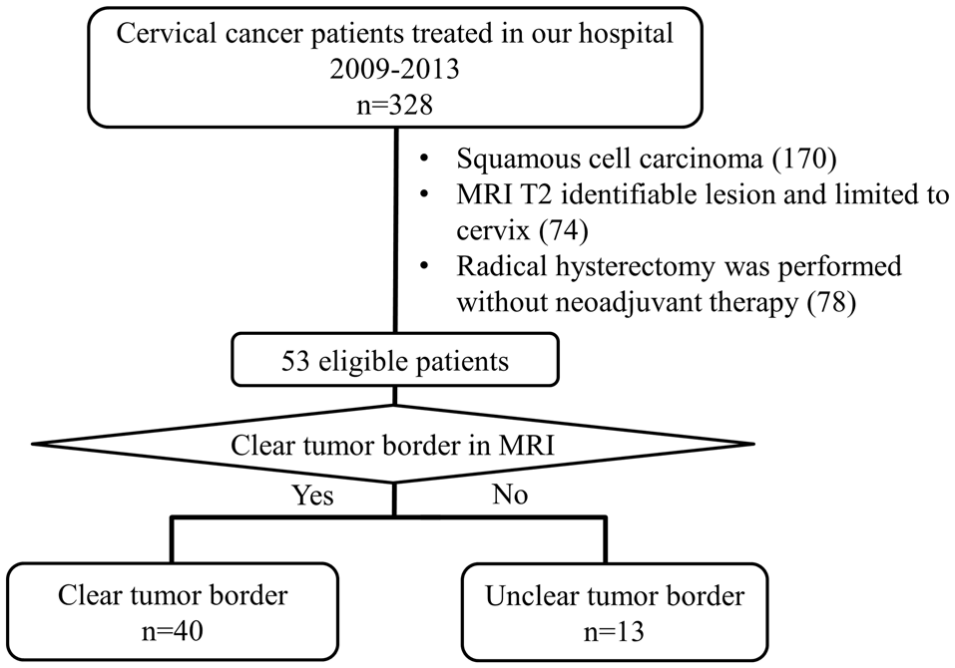
Flow chart of patient selection. MRI, magnetic resonance imaging.

**Figure 2:**
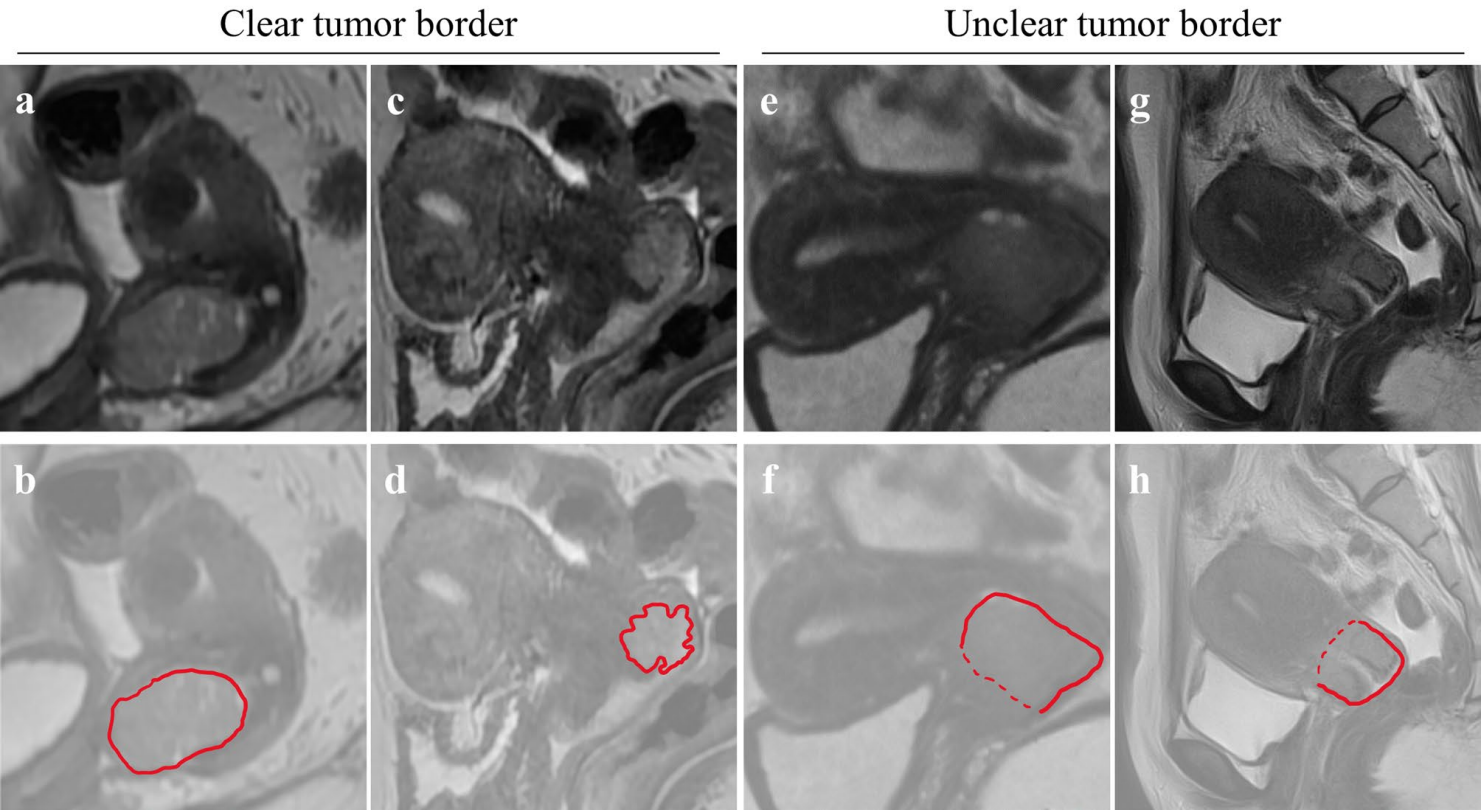
Example of classification based on the tumor border using magnetic resonance imaging. (**a,b**) A 60-year-old patient with a clear tumor border. The solid line indicates the clear borderline of the tumor. (**c,d**) A 46-year-old patient with a clear tumor border. The solid line indicates the clear borderline of the tumor. (**e,f**) A 29-year-old patient with an unclear tumor border. The solid line indicates the clear borderline, while the dotted line indicates the unclear borderline of the tumor. (**g,h**) A 35-year-old patient with an unclear tumor border. The solid line indicates the clear borderline, whereas dotted line indicates the unclear borderline of the tumor.

A total of 328 cervical cancer patients, who underwent primary treatment in Nagoya University Hospital (Nagoya, Japan) between January 2009 and December 2013, were analyzed. Of those, 53 patients diagnosed FIGO stage 1 preoperatively who underwent abdominal radical hysterectomy with identifiable lesions limited in the cervix (according to MRI) were included in the study. Patients with pathologies other than squamous cell carcinoma, those who had received neoadjuvant therapy, as well as those with lesions growing outside the cervix, were excluded (Fig. 1). Subsequently, patients were categorized into two groups based on T2-weighted images, namely clear and unclear tumor borders (Fig. 2). Adjuvant concurrent chemoradiotherapy (CCRT) (50.4 Gy whole pelvic irradiation plus three cycles of 70 mg/m^2^ of cisplatin and 2800 mg/m^2^ of 5-fluorouracil) was administered after the abdominal radical hysterectomy when the risk of recurrence was found based on postoperative pathological findings (LVSI, lymph node metastasis, tumor diameter of > 4 cm or, deep stromal invasion was present). Patient data collected in this study were as follows: age; treatment procedure; overall survival (OS); progression-free survival; imaging findings; pathological findings (e.g., LVSI rate and immunohistochemistry); and site of recurrence. Lymph node metastasis and LVSI were diagnosed pathologically based on surgical specimens. Data collection was approved by the ethics committee of Nagoya University Hospital (approval number: 2019-0106), and approval for an opt-out consent method was provided. The patients provided informed consent for their participation in this study through the website of Nagoya University. The study was performed in accordance with the Declaration of Helsinki.

### Image analysis and definition

The sagittal and horizontal planes of T2-weighted images were used. MRI was performed at multiple institutes; despite differenced in the devices used, 1.5-T scanners were mainly used with a slice thickness of 5.0–6.0 mm. Group were defined as follows: (i) clear tumor border: cancer with a clear outline; (ii) unclear tumor border: cancer with an unclear tumor border between the tumor and normal tissue (Fig. 2). Two gynecologists with > 10-year experience of gynecological cancer treatment interpreted the MRI findings; radiologists also analyzed the data. The gynecologists were blinded to the staging. In case of mismatched categorization, the gynecologists reached a consensus through discussion. Tumor diameter was measured using the T2 sagittal plane, and the maximum diameter was utilized in the analysis.

### Immunohistochemical staining

Paraffin-embedded blocks were sliced into sections (thickness: 4 μm) and mounted onto slides. Following deparaffinization and antigen retrieval, the tissues were incubated with antibodies against vimentin [1:200; #5741; Cell Signaling Technology (CST)] overnight at 4 °C. After washing with phosphate-buffered saline with Tween 20, the tissues were incubated with horseradish peroxidase- conjugated goat anti-rabbit IgG (CST) for 1 h at 37 °C. Finally, the sections were stained using a 3,3'-diaminobenzidine staining solution and counterstained with hematoxylin. All images were captured using a Zeiss Axio Imager.A1 microscope (Carl Zeiss, Tokyo, Japan). Immunoreactivity was evaluated by three gynecologists who were blinded to clinical data. Staining of ≥ 10% cells denoted positivity for vimentin.

### Statistical analysis

The SPSS version 29.0 software (IBM Corp., Armonk, NY, USA) was used to perform the statistical analysis. The chi-squared test and Student t-test were used to evaluate differences in patient characteristics between the groups. To evaluate the power, post-hoc power analysis was performed. Survival curves were plotted using the Kaplan–Meier method, and statistical significance was assessed using the log-rank test. The associations of patient characteristics and MRI subtypes with OS and recurrence-free survival (RFS) were evaluated based on the univariate and multivariate Cox proportional hazards regression models. Results with p-values < 0.05 denoted statistically significant differences.

## Results

Patient characteristics and pathological differences are summarized in Tables 1 and 2, respectively. All patients in the unclear tumor border group (13/13) had LVSI compared with 60% (24/40) in the clear tumor border group. Furthermore, 92% (12/13) of patients exhibited postoperative upgraded staging in the unclear tumor border group compared with 37.5% (15/40) in the clearer border group, and 76.9% (10/13) of patients in the unclear tumor border group exhibited pathological lymph node metastasis versus 15% (6/40) in the clear tumor border group. Because of LVSI and lymph node metastasis, the rate of patients who underwent concurrent chemoradiotherapy as adjuvant therapy was higher in the unclear tumor border group compared with the clear tumor border group (11/13 vs. 24/40, respectively). The recurrence rate also differed between the groups (3/13 vs. 3/40, respectively). The unclear tumor border group had a higher recurrence rate compared with the clear tumor border group (23.1% vs. 7.5%, respectively), both in paraaortic lymph nodes and parenchymal organs.

**Table 1:** Patients' characteristics in each group.

	Clear tumor border (n = 40)	Unclear tumor border (n = 13)	p value
Age, years			
Median	45.4	38.0	0.984*
Range	21.0–77.5	(29.7–68.2)	
FIGO 2018, n			
IB1	23	0	< 0.001**
IB2	0	0	
IB3	2	1	
IIA	5	0	
IIB	4	2	
IIIC1	6	10	
Diameter, cm			
Median	2.5	2.8	0.046*
Range	0.3–5.0	1.6–4.0	
Surgery alone, n			
Yes	16	2	0.104**
%	40.0	15.4	
Surgery + CCRT, n			
Yes	24	11	0.104**
%	60.0	84.6	
Surgical margin positive, n			
Yes	0	0	Not applicable
%	0	0	
Recurrence, n			
Yes	3	3	0.124**
%	7.5	23.1	
Reccurence site, n			
Local	1	0	Not applicable
%	2.5	0	
PAN	1	1	
%	2.5	7.7	
Parenchymal	1	2	
%	2.5	15.4	
Died of disease, n			
Yes	1	3	0.015**
%	2.5	23.1	

*FIGO* International Federation of Gynecology and Obstetrics, *CCRT* concurrent chemo-radiotherapy, *LVSI* lymphovascular space invasion, *PAN* paraaortic lymph node.

*Student t-test, **Pearson's Chi-square test.

**Table 2:** Clinical and tumor pathological characteristics in each group.

	Clear tumor border	Unclear tumor border	p value*	Post-hoc power analysis
Case, n				
Total	40	13		
Pathological LVSI, n			< 0.001	0.927
Positive	24	13		
Negative	16	0		
Pathological lymph node metastasis, n			0.006	0.995
Positive	6	10		
Negative	34	3		
Vimentin, n			0.045	0.522
Positive	18	10		
Negative	22	3		
Recurrence, n			0.124	0.366
Yes	3	3		
No	37	10		
Died of disease, n			0.015	0.632
Yes	1	3		
No	39	10		

*LVSI* lymphovascular space invasion.

*Pearson's Chi-square test.

Table 3 shows the univariate and multivariate analyses for overall survival. Correlation analysis excluded LVSI, lymph node metastasis, and postoperative treatment because they were correlated with tumor boundaries. The tumor border finding was an independent poor prognostic factor for overall survival.

**Table 3:** Uni- and multivariate Cox proportional hazard analysis with overall survival.

	Univariate		Multivariate	
	HR (95% CI)	p-value	HR (95% CI)	p-value
Age, year	0.995 (0.922–1.075)	0.903	0.990 (0.912–1.075)	0.815
Diameter, cm	1.563 (0.608–4.017)	0.354	1.333 (0.429–4.140)	0.620
Unclear tumor border	12.055 (1.245–116.736)	0.032	10.750 (1.080–107.043)	0.043

*MRI* magnetic resonance imaging, *HR* hazard ratio, *CI* confidence interval.

**Figure 3:**
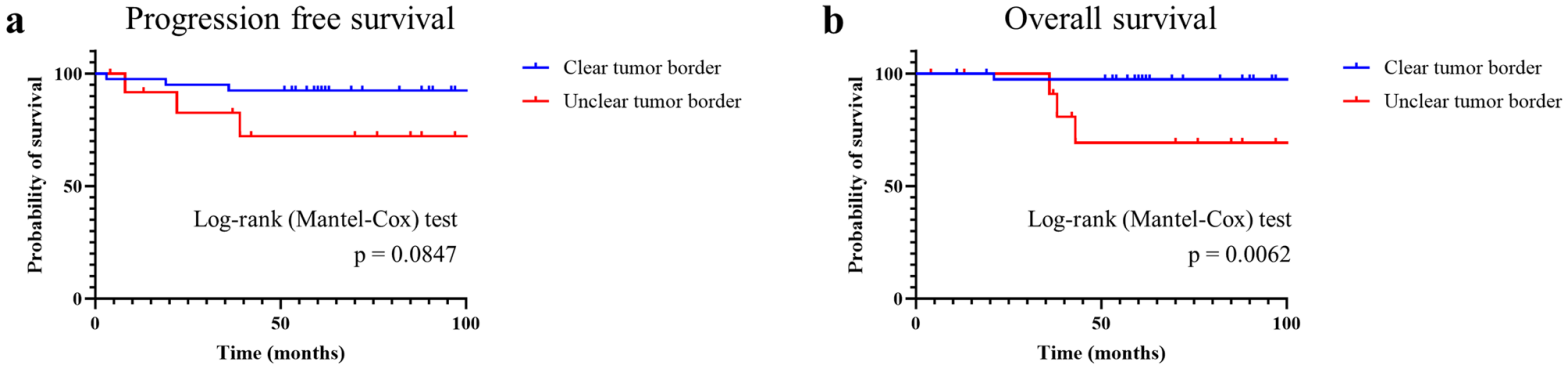
Progression-free survival (**a**) and overall survival (**b**) stratified according to the tumor border.

Figure 3 shows the OS and RFS stratified by the type of tumor border. Although there was no significant difference in RFS (p = 0.0847), the unclear tumor border group was associated with a significantly poorer OS versus the clear tumor border group (p = 0.0062).

**Figure 4:**
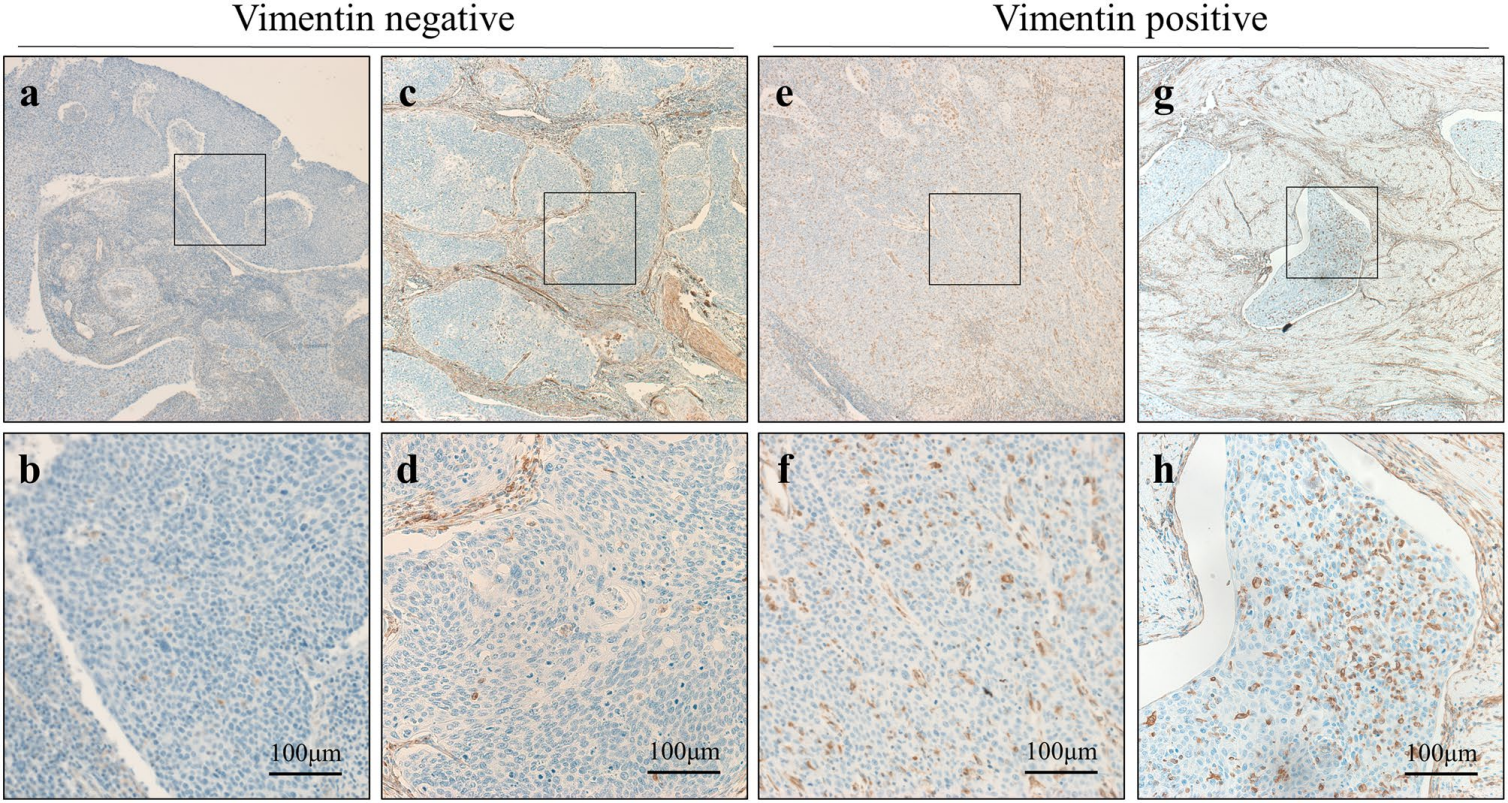
Representative images from the immunohistochemical analysis of vimentin. (**a,b**) Microscopic findings in case 1. Vimentin was poorly expressed. Magnification: ×4 (**a**), ×20 (**b**). (**c,d**) Microscopic findings in case 2. Vimentin was poorly expressed. Magnification: ×4 (**c**), ×20 (**d**). (**e,f**) Microscopic findings in case 3. Tumor cells were diffusely positive for vimentin expression. Magnification: ×4 (**e**), ×20 (**f**). (**g,h**) Microscopic findings in case 4; Tumor cells were diffusely positive for vimentin expression. Magnification: ×4 (**g**), ×20 (**h**).

Figure 4 shows the immunohistochemical findings for vimentin. Based on the results, 37 of the 53 patients exhibited positivity for vimentin expression. As shown in Table 2, positivity for vimentin was significantly higher in the unclear tumor border group compared with the clear tumor border group (p = 0.045).

## Discussion

The results of this study showed that patients with early-stage cervical cancer, for whom operation is considered a treatment option, can be classified using MRI into two groups based on the clarity of the tumor border. Moreover, it was shown that this classification may have prognostic importance. Tumor shape has been reported as a prognostic factor in other malignancies, such as breast cancer, bladder cancer, and meningioma^8–10^. To the best of our knowledge, this study is the first to examine the relationship between tumor border and prognosis in cervical cancer. MRI showed a significantly better diagnostic performance than clinical assessment for both overall staging and evaluation of prognostic factors^11^. As demonstrated in earlier reports, prognostic factors for cervical cancer are tumor size, patient age, stage, lymph node involvement and location, LVSI, histological type, and tumor grade^12–14^. However, some of these factors cannot be assessed unless the patient undergoes surgery. The current study revealed that the tumor border correlates with incidence of LVSI, postoperative upgrade in staging, lymph node metastasis, and distant metastasis. Therefore, these findings could be used to identify cases that would be benefited from radiotherapy and chemotherapy without surgery. Additionally, this evidence may facilitate the process of treatment planning for the preservation of fertility or ovarian function. In patients in whom lymph node metastasis is detected after radical trachelectomy or ovarian-sparing hysterectomy, concurrent chemoradiotherapy is mandatory, and efforts for the preservation of fertility or ovarian function must be abandoned. Hence, radical surgery instead of conservative surgery would be recommended for patients with an unclear tumor border.

Vimentin is a major constituent of the intermediate filament family. It is mainly expressed in mesenchymal cells, and play critical roles in cell adhesion, migration, and signaling^15^. In cancer, vimentin is used as a marker of EMT. EMT is a critical process for cancer metastasis^16^. It has been reported that vimentin is involved in various types of cancer. In prostate cancer, vimentin expression was mainly detected in poorly differentiated tumors and metastatic lesions^17^. In hepatocellular carcinoma, expression of vimentin was mainly associated with metastasis^18^. In non-small-cell lung cancer, vimentin overexpression was identified as an independent prognostic indicator^19^. In cervical cancer, Gilles et al. reported a clear association between vimentin expression and metastatic progression. This conclusion was based on the detection of vimentin in all invasive carcinomas and lymph node metastases, but not in cervical intraepithelial neoplasia 3 (CIN3) lesions^20^. Moreover, Lin et al. reported that vimentin expression is considered as an independent prognostic factor in cervical cancer^7^. Collectively, the currently available data suggests that cervical cancers with unclear tumor borders are associated with higher vimentin expression, lymph node metastasis rate, incidence of LVSI, and recurrence rate versus tumors with clear borders. The potential implication of this study is that cervical cancer with unclear tumor border have a high expression of vimentin and enhanced EMT characteristics such as metastatic potential and invasiveness, and therefore have a high risk of upstaging and lymph node metastasis, and of recurrence and poor prognosis.

There are several limitations in the present study. Firstly, the number of patients with identifiable lesions by MRI who underwent operation was small; therefore, further investigation is warranted to validate the present data. Secondly, due to differences in the prognoses of squamous cell carcinoma and adenocarcinoma, this study focused only on the former type of cancer^21^. Hence, the present findings may not be generalizable to all cervical cancers. Finally, further investigation is warranted to elucidate the mechanism underlying the relationship between vimentin expression and unclear tumor borders.

## Conclusions

According to MRI findings, an unclear tumor border in patients with cervical cancer is linked to poorer prognosis, lymph node metastasis, and distant recurrence of metastasis. MRI findings may act as a predictive factor for postoperative analysis of risk factors, potentially aiding in the choice of treatment options and post-treatment management.

## Data Availability

The datasets used and/or analyzed during the presented manuscript are available from the corresponding author on reasonable request.
